# Mutation in *BMPR2* Promoter: A ‘Second Hit’ for Manifestation of Pulmonary Arterial Hypertension?

**DOI:** 10.1371/journal.pone.0133042

**Published:** 2015-07-13

**Authors:** Rebecca Rodríguez Viales, Christina A. Eichstaedt, Nicola Ehlken, Christine Fischer, Mona Lichtblau, Ekkehard Grünig, Katrin Hinderhofer

**Affiliations:** 1 University Hospital Heidelberg, Centre for pulmonary hypertension of the Thoraxclinic Heidelberg, Heidelberg, Germany; 2 Heidelberg University, Institute of Human Genetics, Heidelberg, Germany; University Hospital Freiburg, GERMANY

## Abstract

**Background:**

Hereditary pulmonary arterial hypertension (HPAH) can be caused by autosomal dominant inherited mutations of TGF-β genes, such as the *bone morphogenetic protein receptor 2 (BMPR2)* and *Endoglin (ENG)* gene. Additional modifier genes may play a role in disease manifestation and severity. In this study we prospectively assessed two families with known *BMPR2* or *ENG* mutations clinically and genetically and screened for a second mutation in the *BMPR2* promoter region.

**Methods:**

We investigated the *BMPR2* promoter region by direct sequencing in two index-patients with invasively confirmed diagnosis of HPAH, carrying a mutation in the *BMPR2* and *ENG* gene, respectively. Sixteen family members have been assessed clinically by non-invasive methods and genetically by direct sequencing.

**Results:**

In both index patients with a primary *BMPR2* deletion (exon 2 and 3) and *Endoglin* missense variant (c.1633G>A, p.(G545S)), respectively, we detected a second mutation (c.-669G>A) in the promoter region of the *BMPR2* gene. The index patients with 2 mutations/variants were clinically severely affected at early age, whereas further family members with only one mutation had no manifest HPAH.

**Conclusion:**

The finding of this study supports the hypothesis that additional mutations may lead to an early and severe manifestation of HPAH. This study shows for the first time that in the regulatory region of the *BMPR2* gene the promoter may be important for disease penetrance. Further studies are needed to assess the incidence and clinical relevance of mutations of the *BMPR2* promoter region in a larger patient cohort.

## Introduction

In different forms of pulmonary arterial hypertension (PAH) as in idiopathic (IPAH), heritable (HPAH), and PAH associated with other conditions (APAH) several mutations in genes of the transforming growth factor beta (TGF-β) superfamily of receptors such as *bone morphogenetic protein receptor 2* (*BMPR2*) gene, *Activin A receptor type II-like 1* (*ACVRL1*, also called *ALK*1) [1], *Endoglin* (*ENG*) [2], and *SMAD9* and *SMAD4* [3] have been found [4].

Mutation of the *BMPR2* gene is the most important causal factor of HPAH and IPAH. Approximately 80% of patients with HPAH and 25% of IPAH-patients [5, 6] carry a mutation in the *BMPR2* gene. Usually only exons are investigated for mutations. However, recently an intronic *BMPR2* mutation has been detected in HPAH-patients showing that probably the frequency of *BMPR2* mutations are even higher than previously detected [7]. Most recently, further rare mutations in genes linked to the BMP signaling pathway have been identified such as *KCNA3* or *KCNA5* mutations in IPAH [8–11], *CAV-1* [12], *BMP9*/*GDF2* in HPAH [13–15], *EIF2AK4* in patients with pulmonary veno-occlusive disease [16, 17].

These findings highlight the importance of BMP signalling for the manifestation of PAH. However, although mutations in the *BMPR2* gene occur in approximately 80–90% of HPAH patients, only about 20–80% of heterozygous gene carriers develop a manifest disease during life-time due to an incomplete age and gender related penetrance [18–20]. The underlying factors of the incomplete penetrance are yet unknown [20].

Most recently, Wang et al. identified two mutations in an IPAH patient, one missense mutation in exon 11 of the *BMPR2* gene and one frameshift mutation in the *KCNA5* gene [21]. The authors hypothesize that the *KCNA5* mutation to be a modifier, which may account for early onset, severity and rapid deterioration of PAH in the index patient. In this study, we worked on a similar hypothesis that additional mutations in the promoter region of the *BMPR2* gene may influence disease penetrance in patients with already known mutation in a TGF-β gene. Depending on the impact of mutations of the *BMPR2* gene a minimum threshold of translated protein may not be reached, which determines whether the disease becomes manifest [22]. Therefore, the aim of this study was to analyze the *BMPR2* promoter region in HPAH families with already diagnosed mutations in TGF-β genes. The promoter region of the *BMPR2* gene was analyzed using direct sequencing in the index patients and family members.

## Materials and Methods

### Study population and design

Two German families (Family 1 and 2) were clinically and genetically examined. Families of index patients with manifest hereditary pulmonary hypertension were studied. A three generation pedigree has been drawn for each family, including 19 and 9 family members, respectively.

All genetically related family members were invited to participate in a clinical and genetic evaluation. After informed consent was obtained, 13 and 7 members, respectively, underwent genetic assessment and counseling. EDTA-blood was taken for genetic analysis. All relatives were residents of a low altitude area and were assessed in Heidelberg, Germany, at an altitude of ~100 meters. Relatives and patients gave their written informed consent to participate in this study. Parents gave written informed consent for any minors participating in this study. The study was approved by the ethical committee at Heidelberg University, Germany.

### Clinical procedures

Clinical procedures consisted of recording the family and medical history, physical examination, routine laboratory parameters including N-type pro brain natriuretic peptide (NT-proBNP), 12-lead ECG, lung function test, arterial blood gases, Doppler-echocardiography, and cardiopulmonary exercise testing. Manifest HPAH was diagnosed according to the current guidelines [23] using right heart catheterization.

### Doppler-echocardiography

Two-dimensional and color-flow guided continuous-wave-Doppler-echocardiographic recordings were obtained using 2.5 MHz Duplex transducers and conventional equipment (Vivid 7, GE Healthcare, Milwaukee, Wisconsin, USA) as described previously [24]. Echocardiographic studies were performed by an experienced cardiac sonographer (EG), who had no knowledge of the molecular genetic data. Doppler-echocardiography and cardiopulmonary exercise testing were performed using a cardiorespiratory diagnosis system (MasterScreen CPX, CareFusion GmbH, Hoechberg, Germany).

### Right heart catheterization

Right heart catheterization (RHC) was performed in two patients. Pressures at rest were recorded using a polygraph (Hellige, Freiburg, Germany) as described before [25]. RHC was done by triple-lumen 7F-Swan-Ganz thermodilution catheters (Edwards Lifesciences, Irvine, CA, USA). Cardiac output (CO) was measured at least in triplicate by thermodilution with a variation of less than 10% between the measured values. The zero reference point for pressure recordings was set at the level of the right atrium in the midaxillary line (phlebostatic axis). All examinations and measurements were performed by the same experienced team. There were no complications.

### Mutation analysis

Genomic DNA was prepared from peripheral blood lymphocytes. In the index patients, the complete coding sequence and exon/intron boundaries of the *BMPR2*, *ACVRL1* (*ALK1*) and *ENG* gene were amplified and analyzed by Sanger sequencing. In a second step, the promoter region of the *BMPR2* gene (up to position c.-1270) was investigated. Family members where tested for mutations that were identified in the index patients by sequencing of the respective genomic regions. Primer sequences and PCR conditions are available upon request. Standard DNA sequencing reactions were performed using version 1.1 of Big Dye terminator cycle sequencing kit (Applied Biosystems Inc., Darmstadt, Germany) and were analyzed on a 3130xl Genetic Analyzer (Applied Biosystems Inc., Darmstadt, Germany). Furthermore, screening for larger *BMPR2* rearrangements was performed with the SALSA Multiplex Ligation-dependent Probe Amplification (MLPA) P093-B2 HHT/PPH1 probe mix kit (MRC-Holland BV, Amsterdam, the Netherlands). Sequence variation nomenclature according to the Human Genome Variation Society (HGVS; www.hgvs.org) was used and refers to the NCBI human *BMPR2* nucleotide sequence (accession number: NM_001204.6) and *ENG* nucleotide sequence (accession number: NM_001114753.2).

## Results

### Clinical characteristics

**Fig 1:**
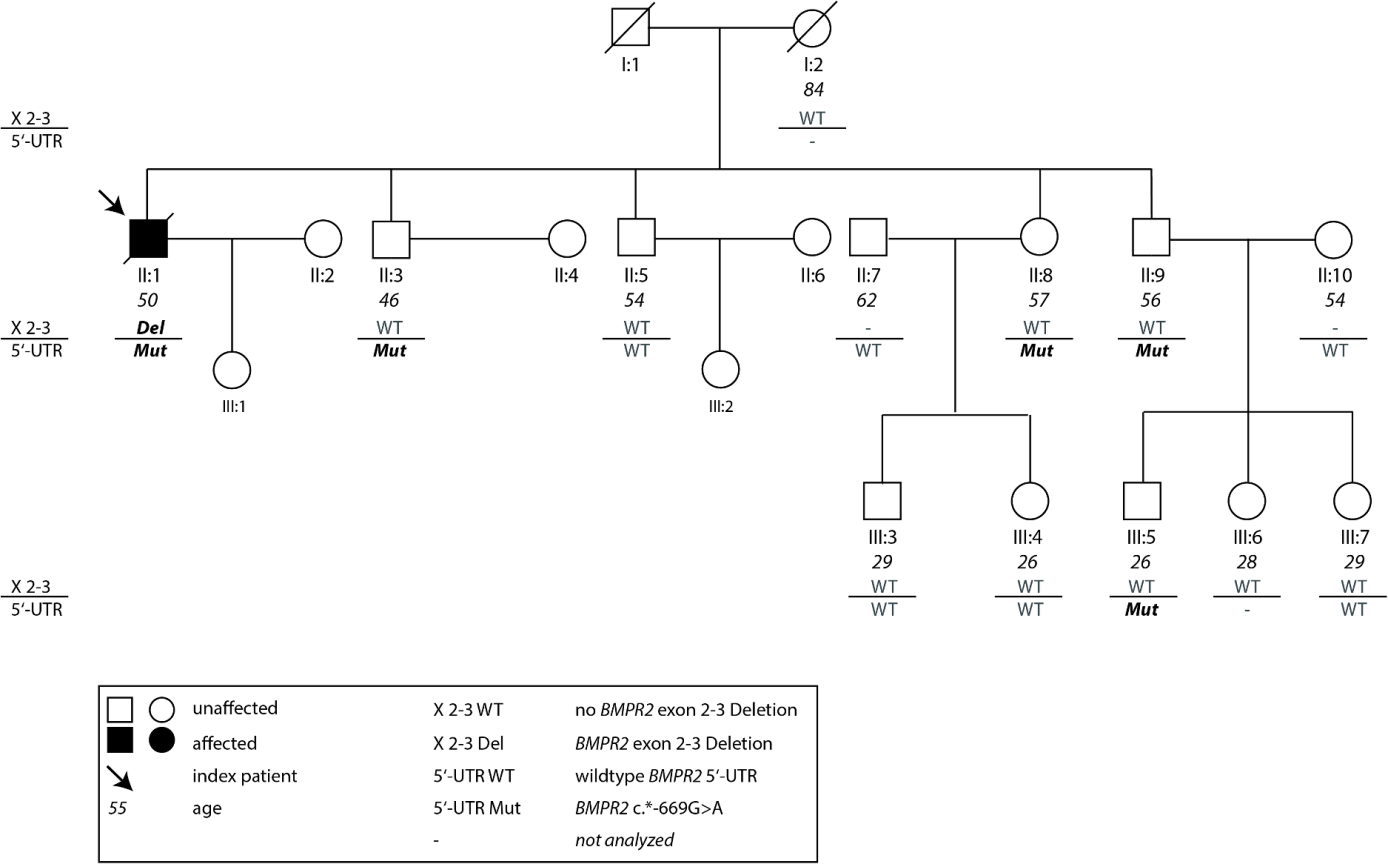
Pedigree of Family 1. The index patient of Family 1 (II:1, arrow) is carrier of the *BMPR2* promoter mutation c.-669G>A [21] and a deletion of exon 2 and 3 of the same gene. The c.-669G>A promoter variant is present in four additional family members (II:3, II:7, II:9, III:5) while the deletion of exon 2 and 3 is unique to the index patient with manifest PAH. Mut: mutation, UTR: untranslated region, WT: wild type, Del: Deletion, X 2–3: exon 2 to 3. The horizontal line separates the two loci in *BMPR2* promoter and *BMPR2* exon 2 to 3.

**Fig 2:**
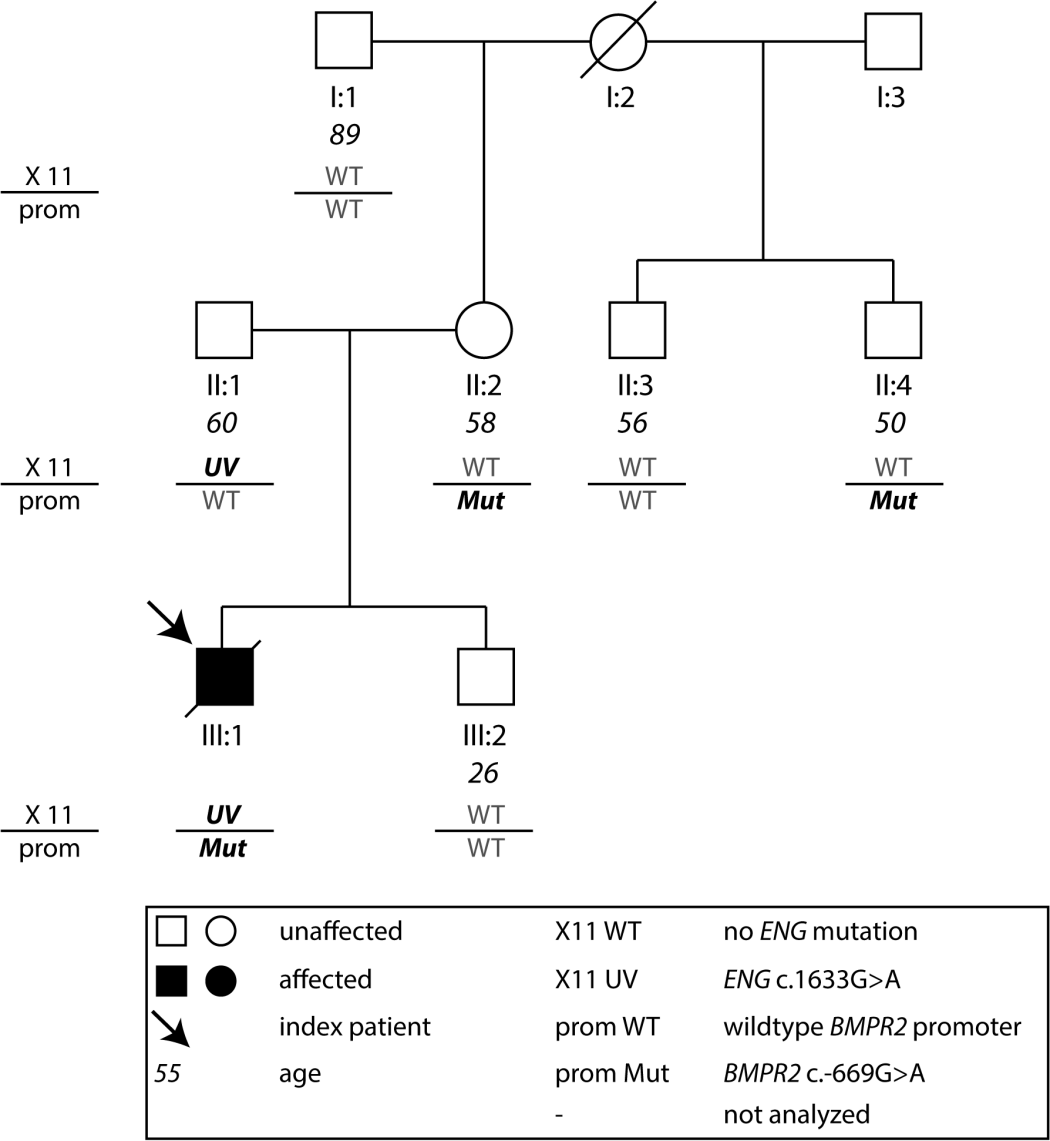
Pedigree of Family 2. The index patient of Family 2 (III:1, arrow) carries the *BMPR2* promoter mutation c.-669G>A [21] and a unclassified variant in the *ENG* gene (c.1633G>A, p.(G545S)). Mut: mutation, UV: unknown variant, UTR: untranslated region, WT: wild type, X 11: exon 11 *ENG*. The horizontal line separates the two loci in *BMPR2* promoter and *ENG* exon 11.

In this study we included 2 index patients initially diagnosed as IPAH and their family members (Figs 1 and 2). The index patient of Family 1 (Fig 1) had symptoms of dyspnea since the age of 27 and was diagnosed at an age of 33 years. At diagnosis he was very severely affected with a mean pulmonary artery pressure (mPAP) of 70 mmHg and a pulmonary vascular resistance of 2000 dyn*s*cm^-5^ (Table 1). The patient was treated with intravenous Iloprost and received a lung transplantation three years after diagnosis. The index patient of Family 2 (Fig 2) was a child of 13 years of age at diagnosis. At age 2 he had an operation of an atrial septal defect. At diagnosis, the mPAP measured with right heart catheterization was 46 mmHg. Pulmonary vascular resistance was 1055 dyn*s*cm^-5^ at rest (Table 1). The patient died with 19 years of age due to PAH and right heart failure. Sixteen family members of both index patients (male = 10, female = 6) were clinically assessed [26–28] and revealed normal findings with normal PAPs and no further signs of pulmonary hypertension (Table 2).

**Table 1:** Clinical characteristics of index patients at diagnosis.

Characteristic	Index patient Family 1	Index patient Family 2
Gender	male	male
Age at diagnosis, years	33	13
Heart rate, min^-1^	89	96
Oxygen saturation, %	90	98
Mean pulmonary artery pressure, mmHg	70	46
Pulmonary vascular resistance, dyn*s*cm^-5^	2000	1055
Cardiac index, l/min/m^2^	1.9	3.6

**Table 2:** Clinical characteristics of family members.

Measurement	Mean* ± SD
Age, years	35 ± 19
Height, cm	167 ± 12
Weight, kg	64 ± 15
Heart rate, min^-1^	72 ± 16
Systemic blood pressure systolic, mmHg	119 ± 19
Systemic blood pressure diastolic, mmHg	76 ± 7
Systemic blood pressure systolic max, mmHg	184 ± 33
Systemic blood pressure diastolic max, mmHg	82 ± 13
Oxygen saturation max exercise, %	94 ± 2
Systolic pulmonary artery pressure rest, mmHg	23 ± 6
Systolic pulmonary artery pressure max, mmHg	48 ± 13
Workload max, Watts	153 ± 55
Heart rate max, min^-1^	160 ± 15
Peak oxygen consumption (VO_2_)/kg, ml/min/kg	29 ± 6
Peak oxygen consumption, ml/min	1927 ± 561
Ventilatory equivalent for carbon dioxide (EqCO_2_) at anaerobic threshold, ml/min	24 ± 4
Oxygen consumption (VO_2_) at anaerobic threshold, ml/min	1515 ± 503
Oxygen pulse, (ml/min)*min^-1^	12 ± 3

*Mean is based on 16 individuals; while maximal blood pressure, peak VO_2_, EqCO_2_, VO_2_ at anaerobic threshold and oxygen pulse are based on 10 individuals

EqCO_2_: ventilatory equivalent for carbon dioxide, max: value at maximal workload, VO_2_/kg: oxygen consumption/kg.

### Genetic analysis

We analyzed the upstream region of the *BMPR2* gene in the two index PAH patients, who carried a mutation or unknown variant in the coding regions of the *BMPR2* and *ENG* gene, respectively (Figs 1 and 2; II:1 in Family 1 and III:1 in Family 2). Both index patients carried the mutation c.-669G>A in the *BMPR2* promoter. This mutation has already been described by Wang and colleagues and functional analysis has been performed, showing reduced expression of *BMPR2* [21].

### Family 1

Family 1 consists of 19 family members. Only the index patient of Family 1 suffered from manifest pulmonary arterial hypertension, which was very severe and diagnosed at young age (Fig 1). The index patient was carrier of the mutation c.-669G>A in the *BMPR2* promoter, which has been previously associated with reduced *BMPR2* expression [21]. Moreover, he had a deletion of exon 2 and 3 of the *BMPR2* gene (c.77-?_418+?del; Fig 1). This deletion leads to the loss of a large part of the extracellular ligand binding domain encoded by exons 1 to 3 [29]. Other deletions of exon 2 and/or 3 have been previously described in PAH patients [30, 31]. The deletion of exon 2 and 3 of the *BMPR2* gene was not present in the index patient’s mother. Due to insufficient amounts of DNA she could not be tested for the mutation in the promoter. Furthermore, we had no DNA of the father and could thus not test him for the two mutations. Nevertheless, we assume the deletion was transmitted from the father and the promoter mutation was transmitted from the mother, because she did not carry the deletion. However, we have to take into consideration the possibility of a *de novo* occurrence of one or both mutations.

The c.-669G>A promoter mutation was present in four additional healthy family members while the deletion of exon 2 and 3 was unique to the index patient with manifest PAH. None of the family members with the c.-669G>A mutation carried the *BMPR2*-deletion and none were clinically affected.

### Family 2

Family 2 consists of 9 family members. Similarly as in Family 1, only the index patient was very severely affected by PAH. He harbored the same *BMPR2* promoter mutation c.-669G>A as described for Family 1 (Fig 2). Additionally, the patient carried a variant in the *ENG* gene (c.1633G>A, p.(G545S)), which has been described earlier [32]. In the previous study the p.(G545S) variant was classified as a polymorphism because it was also present in the healthy mother (62 years) of the index patient and the index patient carried an additional frameshift mutation that was assumed to be disease causing [32]. Nevertheless, the authors suggested the possibility of a disease modifying effect for p.(G545S). Functional analyses for the latter variant have not been performed yet and prediction tools are inconclusive: PolyPhen-2 “probably damaging” [33], SIFTS “tolerated” [34], Align GVGD “C0” (less likely to interfere with function) [35] and MutationTaster “disease causing” [36]. Together with the findings in this study it is possible that the variant has a disease-modifying effect. In our family the index patient inherited one respective variant from each parent, both unaffected by PAH. The mother and a half-brother of the mother were also carriers of the c.-669G>A *BMPR2* promoter mutation without clinical signs of PAH. This is in concert with the findings of Family 1, where we found that the described promoter variation does not lead to PAH when observed on its own.

## Discussion

In this study we describe for the first time that only those family members carrying a *BMPR2* promoter mutation and an additional mutation/variant in a TGF-β gene develop PAH, whereas their family members with only one mutation were not affected. Our findings are in line with the hypothesis that two mutations are contributing to the development and manifestation of PAH and have a penetrance-modifying effect. We hypothesize that the identified germline mutation in the regulatory region of the *BMPR2*gene as a “second-hit” leads to a very severe clinical phenotype at young age in the affected family members.

### Mutations in the promoter region of *BMPR2* gene

Regulatory regions of TGF-β genes are not a major focus in PAH diagnostics so far, though they might harbor disease causing mutations. To our knowledge, only two previous studies describe HPAH families, in which a mutation in the *BMPR2* promoter was identified in an index patient [21, 37]. By measuring the relative abundance of mutant versus wild-type transcripts in leukocyte cDNA obtained from the patient, it was shown that the variant c.-944/5GC>AT leads to a significant decrease of the *BMPR2* mRNA expression [37]. Furthermore, Wang and colleagues showed that the c.-669G>A promoter mutation caused a disruption of a SP3 transcription factor-binding site [21]. As a consequence, the mutated promoter sequence showed significantly decreased transcriptional activity in luciferase assays in comparison to the wild-type promoter sequence. These studies demonstrate that the upstream region of the *BMPR2* gene is of yet underestimated importance in PAH diagnostics.

### Second-hit mutation may influence disease penetrance

The results of this study show that the interplay of different mutations or variants in the same pathway may be the critical difference between disease manifestation and healthy carriers. A similar pattern was also observed in two studies of American PAH families, where age of onset and penetrance was influenced by a second mutation, additional to *BMPR2*, in the *TGFB1* gene in the first family and in the TGF-β regulating gene *THBS1* in the second family [38, 39]. This hypothesis has also been suggested by the study of Wang et al. [9]. Similarly, variations in the regulatory region of the gene, as observed in the promoter in this study, may impact disease penetrance. It is interesting to note that all healthy family members carrying a variant or mutation in *BMPR2* or *ENG* were heterozygotes. Thus, sufficient protein might be produced in carriers of one mutation or variant by the intact allele obtained from the other parent preventing disease manifestation. In contrast, in affected patients the modifier gene may lead to a strong signaling reduction and disease manifestation. Phillips et al. [38] showed that *TGFB1* single nucleotide polymorphisms (SNPs) modulate the age at diagnosis and penetrance of HPAH in *BMPR2* mutation heterozygotes. This modulation is an example of synergistic heterozygosity and likely functions by affecting TGF-β /BMP signaling imbalance.

### Modifier genes in other hereditary diseases

In other hereditary diseases further modifying genes have been identified as well. For example, the long QT syndrome (LQTS) has been considered classically an autosomal dominant genetic disorder, with heterozygous mutations in the three major LQTS-susceptibility genes (*KCNQ1/LQT1*, 30%–35%; *KCNH2/LQT2*, 25%–30%, and *SCN5A/LQT3*, 5%–10%) marked incomplete penetrance and variable expressivity [40–42]. It has been described that penetrance and expressivity in *KCNQ1* mutation-positive subjects was modified by the common *KCNE1-D85N* polymorphism [43–45]. Thus, it appears that common amino acid-altering genetic variation may also serve as genetic modifiers of LQTS disease severity regardless of whether the second hit occurs within or outside the gene harboring the primary disease causing mutation.

Other well-studied examples are the hair disease monilethrix (*KRT86*) [46] and different types of retinis pigmentosa (*PRPF8* and *PRPF31*) [47, 48].

### Change in genetic screening methods might be useful

Today, standard genetic screening methods focus on sequencing of coding regions of the *BMPR2*, *ALK1*, *Endoglin*, *SMAD9*, *CAV1*, *KCNK3* and *EIF2AK4* genes [49]. Although HPAH is widely accepted as a monogenetic disease with autosomal dominant inheritance, only 20–80% of *BMPR2* mutation carriers will develop the disease due to an incomplete age and gender related penetrance [18, 19]. It is however common to stop mutation screening as soon as one mutation has been identified in the index patient. Thus, additional mutations might be missed and those second mutations with a major impact on the manifestation of PAH could be overlooked. Therefore, we suggest thorough screening of further genes in patients, in whom one disease-causing mutation has already been identified to obtain a full picture of genetic factors influencing disease manifestation. This will be facilitated with the advent of highly parallel next generation sequencing technologies. In this manner, the further causes for the reduced penetrance could be revealed and earlier identification of high risk family members may be achieved.

## Conclusion

The results of the present study, led us to the hypothesis that family members with two mutations have a higher risk to develop manifest PAH than family members carrying only one mutation or variant. To complement our knowledge of the genetic factors influencing disease manifestation, we propose thorough screening of index patients with mutation-carrying albeit healthy family members.
